# Malnutrition in Maintenance Dialysis: From Metabolic Disorder to Multisystem Complications

**DOI:** 10.3390/jcm15197451

**Published:** 2026-09-24

**Authors:** Keren Cohen-Hagai, Moshe Shashar, Kamyar Kalantar-Zadeh

**Affiliations:** 1 Department of Nephrology and Hypertension, Meir Medical Center, Kfar Saba 4428164, Israel; 2 Gray Faculty of Medical and Health Sciences, Tel Aviv University, Tel Aviv 6997801, Israel; 3 Renal Section, Laniado Medical Center, Netanya 4244916, Israel; 4 Division of Nephrology, Hypertension and Kidney Transplantation, Harbor-UCLA Medical Center and the Lundquist Institute, Torrance, CA 90502, USA; 5 Tibor Rubin Veterans Affairs Medical Center, Long Beach, CA 90822, USA

**Keywords:** malnutrition, protein-energy wasting, malnutrition–inflammation syndrome, dialysis complications

## Abstract

Malnutrition and protein-energy wasting (PEW) are closely related yet distinct clinical entities. While malnutrition broadly refers to nutritional deficits arising primarily from inadequate dietary intake, PEW is a complex syndrome specific to chronic kidney disease characterized by the progressive loss of body protein mass and energy reserves driven by systemic hypercatabolism, uremic toxicity, chronic inflammation that cannot be corrected by dietary intake alone. Nutritional impairment remains one of the most prevalent and clinically significant complications of end-stage kidney disease (ESKD), affecting a substantial proportion of patients receiving maintenance hemodialysis and peritoneal dialysis despite advances in dialysis technology and multidisciplinary care. Traditionally, nutritional impairment in dialysis was evaluated through isolated surrogate markers such as serum albumin or body weight. However, these conventional measures capture only a fraction of the underlying biological complexity and are heavily confounded by non-nutritional factors, including systemic inflammation and volume overload. Recognizing the intertwined contributions of uremic milieu, inflammation, oxidative stress, and hypercatabolism led to a paradigm shift toward composite nutritional assessment tools, most notably the Malnutrition–Inflammation Score (MIS), which integrates dietary, anthropometric, clinical, and biochemical parameters to comprehensively quantify PEW severity and systemic risk. Current evidence supports a broader conceptual framework in which nutritional status is recognized as an important clinical correlate and potential contributor to metabolic health and multisystem complications, including immune dysfunction, cardiovascular disease, frailty, hospitalization, and all-cause mortality. Consequently, nutritional impairment in dialysis should be viewed as a multifactorial clinical condition requiring comprehensive, multi-domain assessment rather than reliance on isolated biomarkers. This review examines the systemic consequences of malnutrition and PEW in maintenance dialysis patients, highlighting emerging evidence linking nutritional deficits with host immune impairment, cardiovascular disease, and adverse clinical outcomes.

## 1. Introduction

Nutritional disorders are common and clinically important complications of end-stage kidney disease (ESKD). Malnutrition and protein-energy wasting (PEW) represent distinct but overlapping concepts [1,2,3]. While malnutrition broadly refers to nutrient depletion resulting strictly from inadequate dietary intake, PEW represents a distinct, complex syndrome specific to chronic kidney disease (CKD) [1]. Driven by the convergence of chronic uremic inflammation, systemic hypercatabolism, metabolic acidosis, endocrine resistance, oxidative stress, and dialytic nutrient losses, PEW involves the progressive depletion of skeletal muscle mass and adipose fuel reserves that cannot be corrected by dietary intake alone [2]. In maintenance dialysis patients, pure malnutrition rarely exists in isolation; rather, inadequate dietary intake and uremic anorexia frequently coexist with and aggravate PEW, forming an intertwined immunometabolic disorder [3,4,5].

Accumulating evidence suggests that nutritional depletion in ESKD extends beyond its role as a marker of illness severity and may be associated with multisystem complications and organ dysfunction [4,6]. Furthermore, malnutrition has been associated with poorer health-related quality of life, including reduced physical performance, greater fatigue, limitations in daily activities, and impaired cognitive and psychological well-being [2,6,7,8,9,10,11]. Despite these far-reaching effects, nutritional deterioration remains underrecognized or oversimplified in routine practice, where assessment often relies on isolated biomarkers such as serum albumin or static body weight [4].

While previous literature has addressed the epidemiology, diagnosis, and management of PEW, the potential consequences of malnutrition and PEW across multiple organ systems have not always been considered together. This review brings together the available evidence on these associations and discusses the potential clinical complications of malnutrition and PEW in patients receiving dialysis.

The literature was identified through searches of major medical databases, with a primary search conducted in PubMed using the keywords “malnutrition in maintenance dialysis” and “complications” for publications from 2000 through 2026. The PubMed search was conducted in September 2026. This search yielded 359 records. Studies were considered for inclusion if they were directly relevant to malnutrition, protein-energy wasting, or their clinical complications in patients receiving maintenance dialysis. The search was used to identify relevant literature rather than as a formal systematic search strategy. Priority was given to contemporary clinical practice guidelines and consensus statements, as well as key primary studies directly relevant to the topics addressed in this review. Key earlier studies that established important concepts in the field were also considered. Additional relevant publications were identified through screening of reference lists of selected articles. Given the narrative nature of the review, no formal systematic-review methodology, risk-of-bias assessment, or quantitative synthesis was performed.

## 2. Epidemiology

Reported prevalence of PEW ranges from <2% in CKD stages 1–2 to approximately 11–54% in CKD stages 3 to 5, increasing to 28–54% among patients receiving maintenance dialysis [12]. The rising prevalence of PEW across the CKD continuum is driven by a complex, multi-factorial pathophysiology. Rather than resulting solely from inadequate dietary intake or uremic toxin retention, PEW arises from the interplay of chronic systemic inflammation, accelerated muscle proteolysis, metabolic acidosis, endocrine resistance, uremic anorexia, and dialysis-related nutrient losses [2,3]. For instance, hemodialysis sessions induce an estimated loss of 6 to 12 g of free amino acids per treatment, whereas peritoneal dialysis involves continuous peritoneal losses of 5 to 15 g of protein and amino acids daily [2,3]. Additionally, chronic systemic inflammation, present in 30% to 60% of North American and European dialysis patients, promoting skeletal muscle breakdown and suppressing hepatic albumin synthesis [4,5]. Consequently, both the prevalence and severity of PEW increase progressively with declining glomerular filtration rate and advancing dialysis vintage.

The reported prevalence estimates vary substantially, largely because studies have used different definitions and assessment tools and have evaluated related but distinct conditions, including malnutrition, PEW, and nutritional risk. Estimates also vary according to patient characteristics, dialysis modality, dialysis vintage, and the clinical setting [12,13,14].

## 3. Definition and Terminology

The terminology used to describe nutritional, metabolic, and functional impairment in patients with ESKD has evolved considerably over the past decades [1,2,3,15]. In both nephrology literature and clinical practice, terms such as malnutrition, *PEW,* cachexia, sarcopenia, and frailty are frequently used interchangeably despite representing distinct, albeit overlapping, clinical entities [1,2]. This conceptual ambiguity has contributed to substantial variability in reported prevalence rates, heterogeneous study cohorts, and challenges in comparing clinical outcomes across trials [12,16]. A precise distinction among these syndromes is therefore essential for elucidating their underlying pathophysiology, selecting valid diagnostic tools, and implementing targeted therapeutic interventions [2,3,6] [see Table 1].

To establish clinical clarity, a fundamental distinction must be drawn between pure malnutrition and PEW [2]. Malnutrition refers strictly to an imbalance arising from inadequate dietary protein and energy intake relative to requirements, a state that can be corrected by nutrient repletion alone [2]. In contrast, PEW, a term established by the International Society of Renal Nutrition and Metabolism (ISRNM), describes a complex immunometabolic syndrome specific to kidney disease, characterized by the progressive loss of skeletal muscle mass and adipose fuel reserves [2,3]. Propelled by chronic uremic inflammation, systemic hypercatabolism, metabolic acidosis, insulin and growth hormone resistance, and dialytic nutrient losses, PEW involves active muscle proteolysis that cannot be reversed by dietary intake alone [2,3,17]. In maintenance hemodialysis and peritoneal dialysis populations, pure malnutrition (e.g., driven by uremic anorexia or strict dietary restrictions) frequently coexists with and aggravates PEW, creating a self-perpetuating cycle of structural and metabolic deterioration [1,2,18].

Cachexia represents the most severe, extreme manifestation of PEW, characterized by profound involuntary weight loss, uncompensated catabolism, and cytokine-mediated systemic inflammation [2,12,19]. Sarcopenia, by contrast, is specifically defined by the progressive depletion of skeletal muscle mass, muscle strength, and physical performance; while it is highly prevalent in dialysis patients, it reflects an impairment of muscle health that can occur independently of overall adiposity or weight loss [2,20,21,22]. Frailty constitutes a broader, multidimensional syndrome of diminished physiological reserve and heightened vulnerability to stressors, encompassing physical weakness, slow gait, exhaustion, and functional decline [3]. Although malnutrition, PEW, cachexia, sarcopenia, and frailty often co-occur and synergistically reinforce each other, they are not synonymous [2,13]. Rather, they represent interconnected yet distinct clinical syndromes that collectively drive the high burden of disability, infection, cardiovascular disease, hospitalization, and mortality observed in maintenance dialysis patients [2,4,23].

## 4. Pathogenesis

Malnutrition and PEW are highly prevalent in the dialysis population and result from a multifactorial and interrelated pathophysiology involving reduced intake, increased catabolism, nutrient losses, endocrine dysregulation, and gut–immune interactions [2,20]. Patients with kidney failure commonly experience anorexia and thus PEW driven by uremic toxin accumulation, altered taste perception, chronic inflammation, depression, and medication burden, all of which contribute to inadequate dietary intake (Figure 1) [4,5]. In parallel, a persistent catabolic state is promoted by the dialysis procedure itself, metabolic acidosis, systemic inflammation, recurrent infections, and frequent hospitalizations [24]. These processes are further amplified by endocrine disturbances characteristic of advanced CKD, including insulin resistance, growth hormone resistance, testosterone deficiency, and vitamin D deficiency. In addition, gut dysbiosis with microbial shifts and endotoxemia contributes to chronic inflammation and further metabolic derangements [20,24].

In maintenance hemodialysis (HD), procedure - and treatment-related factors represent a major contributor to PEW. During each standard HD session, significant dialytic clearance of essential substrates occurs, including a measurable loss of free amino acids, alongside variable amounts of peptides, water-soluble vitamins (such as vitamin C, folate, and B-complex vitamins), and essential trace elements (e.g., zinc and selenium). Recurring three times weekly, these cumulative dialytic losses create a persistent bioenergetic drain that spontaneous oral intake frequently fails to replenish1more_horiz [24,25].

Compounding these direct physical losses, the HD procedure itself triggers repetitive bioenergetic and catabolic stress. Contact between patient blood and the non-biological surfaces of dialyzer membranes, particularly those with lower biocompatibility, and arterial-venous blood tubing activates the complement cascade, circulating monocytes, and neutrophils. This bioincompatibility response, along with potential exposure to dialysate impurities or the back-filtration of endotoxins, induces the rapid production and systemic release of proinflammatory cytokines [4,24,25,26]. The systemic inflammation, often acting synergistically with uremic metabolic acidosis, directly upregulates the ATP-dependent ubiquitin-proteasome pathway in skeletal muscle, driving heavy myofibrillar proteolysis and suppressing hepatic albumin synthesis. When superimposed on a background of uremic anorexia, delayed gastric emptying, and prescribed restrictive diets (e.g., potassium and phosphate restrictions), these dialytic nutrient drains and hypercatabolic stimuli drive the patient into a chronic negative nitrogen and energy balance, thereby systematically reinforcing the progressive loss of lean body mass and visceral protein reserves [1,4,5,24].

In peritoneal dialysis (PD), procedure-specific metabolic and physical factors drive a distinct pathway toward PEW [25]. Unlike the intermittent nutrient clearance seen in HD, nutrient depletion in PD is characterized by continuous, daily trans-peritoneal losses across the peritoneal membrane. Stable PD patients lose an estimated 5 to 10 g of protein and free amino acids daily into the spent dialysate, with albumin accounting for the vast majority of this protein drain [27]. During acute or subclinical episodes of peritonitis, peritoneal membrane permeability increases markedly, escalating daily protein losses even further and requiring increased dietary protein targets (≥1.5 g/kg/day) to prevent acute nitrogen collapse [1,2,3]. Furthermore, patients with a high peritoneal transport status experience greater solute transport rates and heightened peritoneal protein leakage, placing them at an elevated baseline risk for persistent hypoalbuminemia and skeletal muscle wasting [25].

Compounding these direct protein losses, the continuous nature of PD presents unique metabolic and appetite-suppressing challenges. Exposure to glucose-based peritoneal dialysate solutions results in the systemic absorption of approximately 50 to 200 g of glucose per day [28]. This continuous peritoneal glucose absorption provides a constant non-protein caloric load that frequently induces abdominal distension, delayed gastric emptying, and early satiety. This “dialytic anorexia” significantly reduces spontaneous oral food and protein intake. Systemically, chronic glucose loading promotes hyperinsulinemia, exacerbates uremic insulin resistance, and alters adipokine regulation (such as elevated leptin levels), driving metabolic dysregulation where patients may gain fat mass while progressively losing lean skeletal muscle, a phenotype recognized as sarcopenic obesity [29].

In addition, PD is associated with localized and systemic inflammatory pathways that exacerbate muscle breakdown. Continuous exposure of the peritoneal cavity to bioincompatible dialysate solutions, characterized by low pH, high osmolality, and elevated concentrations of glucose degradation products incites local peritoneal inflammation and mesothelial cell injury [3,27]. This local response, combined with the presence of the peritoneal catheter as a chronic foreign body, latent catheter-site infections, and recurrent peritonitis, stimulates local and systemic monocyte activation. Proinflammatory cytokines released into circulation downregulate hepatic albumin synthesis and, in combination with metabolic acidosis accelerate muscle proteolysis [3,27].

Together, continuous peritoneal protein losses, glucose-induced anorexia, metabolic dysregulation, and bioincompatibility-driven inflammation create a chronic negative nitrogen balance. When spontaneous dietary protein intake fails to meet or exceed recommended guidelines (1.0–1.2 g/kg/day for stable PD patients), these synergistic mechanisms place PD patients at sustained risk for progressive, uncompensated PEW [1,2].

## 5. Immunological Consequences

Immune dysfunction is a well-recognized complication of advanced kidney failure and reflects the combined effects of uremia, chronic inflammation, metabolic disturbances, and other host-related factors [30]. Nutritional impairment and PEW may further contribute to impaired immune competence and reduced physiological reserve [30,31,32,33,34,35].

The innate immune response is substantially impaired in dialysis patients. Neutrophils exhibit defective chemotaxis, reduced phagocytic capacity, and an impaired oxidative burst, limiting their ability to eliminate invading pathogens effectively. Monocyte and macrophage function is also altered, with impaired antigen presentation and dysregulated cytokine production that contributes simultaneously to ineffective host defense and chronic low-grade inflammation [34,35]. In addition, the dialysis procedure itself may transiently activate innate immune pathways through blood–membrane interactions, further promoting oxidative stress while ultimately contributing to immune dysfunction [30,35,36,37].

Adaptive immunity is similarly compromised. Dialysis patients frequently develop lymphopenia, particularly affecting naïve T-cell populations, accompanied by thymic involution and reduced thymic output [38,39]. Chronic antigenic stimulation and persistent inflammation promote T-cell exhaustion, characterized by impaired proliferation, reduced cytokine production, and diminished cytotoxic activity. B-cell number and function are likewise reduced, resulting in impaired antibody production and weakened humoral immunity. Collectively, these abnormalities increase susceptibility to bacterial and viral infections while reducing long-term immunological memory [38,39,40].

These immune abnormalities resemble accelerated immunosenescence, a hallmark of advanced kidney failure characterized by premature aging of the immune system. Immunosenescence is closely linked to frailty, sarcopenia, and protein-energy wasting, and is perpetuated by chronic inflammation (“inflammaging”), oxidative stress, and nutritional deficiencies [41]. Frail and patients with PEW demonstrate greater immune dysfunction, which further increases the risk of infection, hospitalization, and mortality, creating a vicious cycle in which inflammation promotes malnutrition and malnutrition further exacerbates immune impairment [41,42,43].

Impaired immune competence also translates into reduced vaccine efficacy. Patients receiving dialysis exhibit lower seroconversion rates and more rapid waning of protective antibody titers following vaccination compared with the general population [44,45]. This phenomenon has been well documented for hepatitis B vaccination, where hypoalbuminemia and markers of PEW are associated with a diminished serological response [46,47]. Similar findings have been reported following COVID-19 vaccination, with malnourished and frail patients demonstrating weaker humoral and cellular immune responses and reduced durability of protection. Improving nutritional status may therefore enhance vaccine responsiveness and represents an important adjunct to immunization strategies in patients with ESKD [48,49,50,51,52,53,54,55].

Micronutrient and vitamin deficiencies in dialysis patients may also affect immune function and other physiological processes. Targeted supplementation of these nutrients may offer potential clinical benefits, with studies of omega-3 fatty acid supplementation providing an example of an intervention associated with improved clinical outcomes, including lower mortality, in patients receiving dialysis [56]. Zinc is another nutrient of particular relevance to immune function, playing critical roles in both innate and adaptive immunity through effects on neutrophil activity, lymphocyte development, and cytokine signaling. Selenium functions as an essential antioxidant and supports T-cell and natural killer cell activity, while deficiencies in vitamins D, C, B6, B12, and folate impair multiple aspects of immune regulation [57,58]. Because several water-soluble vitamins and trace elements are lost during dialysis or are inadequately consumed, careful assessment and appropriate replacement of micronutrient deficiencies may help optimize immune function, although routine supplementation beyond established deficiencies remains an area of ongoing investigation [58,59,60,61].

## 6. Infection

Infections are a major cause of morbidity, hospitalization, and mortality among patients receiving dialysis, although their relative contribution to mortality varies across populations and healthcare settings. In a large Australia and New Zealand registry cohort, infection accounted for 12% of deaths among patients receiving hemodialysis or peritoneal dialysis. Although infection-related mortality declined over time, it remained more than 20-fold higher than in the age-matched general population [62]. The increased susceptibility to infection in patients receiving dialysis reflects the complex interplay of multiple factors, including uremia-related immune dysfunction, repeated exposure to healthcare environments, invasive vascular or peritoneal access, and the high prevalence of PEW [30,33,34]. Nutritional impairment and PEW have been associated with an increased risk of infectious complications, although their independent contribution is difficult to disentangle from coexisting inflammation, comorbidity, and kidney failure–related immune dysfunction [30,33,34].

Inadequate protein and energy intake may adversely affect wound healing, epithelial and mucosal barrier integrity, and the synthesis of complement proteins and other components of the innate immune response. These alterations may contribute to impaired host defense and susceptibility to infection. Accordingly, malnutrition in patients receiving dialysis has been associated with a higher burden of infectious complications, prolonged hospitalization, and increased infection-related mortality, although these associations are likely influenced by the multiple comorbidities and other risk factors characteristic of the dialysis population [20,63,64,65].

The spectrum of infections differs between dialysis modalities. In hemodialysis, vascular access infections remain one of the most important complications, particularly among patients dialyzing through central venous catheters, where local infection may rapidly progress to bacteremia and sepsis. Impaired wound healing associated with malnutrition delays recovery following vascular access procedures and increases the risk of exit-site infections and poor tissue repair. Bloodstream infections frequently originate from infected vascular access and are associated with substantial morbidity, including infective endocarditis, metastatic infections, and septic shock. Pneumonia also represents an important cause of hospitalization and mortality in the hemodialysis population. Whether nutritional impairment independently contributes to susceptibility or clinical outcomes remains difficult to establish because of multiple coexisting risk factors [63,64].

In peritoneal dialysis, peritonitis remains the hallmark infectious complication and is a major cause of technique failure and transition to hemodialysis. Nutritional impairment contributes to the development and persistence of peritoneal infections through several mechanisms, including defective local immune responses, reduced complement production, impaired leukocyte function, and delayed tissue repair. Hypoalbuminemia has consistently been identified as an independent predictor of peritonitis, recurrent infections, and infection-related mortality [65,66,67]. Infectious episodes, including vascular access infections, peritonitis, pneumonia, bacteremia, and sepsis, may further increase inflammatory and catabolic activity and thereby contribute to worsening nutritional status. Conversely, PEW and its associated alterations in immune and metabolic function may increase susceptibility to infection. Thus, infection and PEW may interact in a bidirectional manner, potentially reinforcing nutritional and inflammatory disturbances in patients receiving dialysis [6].

## 7. Cardiovascular Consequences

In addition to infections, cardiovascular disease remains the leading cause of death in patients receiving dialysis, and inflammation–malnutrition is increasingly recognized as an active contributor rather than merely a marker of poor prognosis [4]. Although hypoalbuminemia has traditionally been used as the principal nutritional biomarker associated with cardiovascular risk, the detrimental effects of PEW extend far beyond low serum albumin concentrations. Nutritional impairment and PEW are closely associated with chronic inflammation, oxidative stress, and adverse cardiovascular outcomes in patients with kidney failure; however, the causal contribution of these interconnected processes is difficult to establish [68,69,70]. Reduced antioxidant defenses and micronutrient deficiencies further amplify oxidative damage, leading to impaired nitric oxide bioavailability, endothelial activation, and progression of vascular disease [4,5].

The interplay between PEW, malnutrition and inflammation may contribute to the development and progression of cardiovascular complications in ESKD. Chronic inflammation stimulates protein catabolism while simultaneously promoting endothelial dysfunction, vascular calcification, and atherosclerotic plaque development [71,72,73]. This relationship is encompassed by Malnutrition–Inflammation–Atherosclerosis syndrome, a conceptual framework describing the reciprocal interaction between PEW, persistent inflammation, and accelerated cardiovascular disease [74]. Rather than acting as independent processes, PEW, malnutrition and inflammation reinforce one another, resulting in progressive vascular injury, increased arterial stiffness, and diffuse calcification of both the intimal and medial arterial layers. Consequently, patients with PEW experience substantially higher rates of ischemic cardiovascular events and cardiovascular mortality than well-nourished individuals [74,75,76,77,78,79].

Beyond vascular disease, malnutrition contributes directly to adverse cardiac remodeling. Loss of skeletal muscle mass is frequently accompanied by myocardial wasting, impaired myocardial energetics, and reduced cardiac reserve. Chronic inflammation and metabolic abnormalities promote left ventricular hypertrophy, myocardial fibrosis, and progressive ventricular dysfunction, increasing the risk of both heart failure and malignant ventricular arrhythmias. These structural and functional alterations help explain the strong association between PEW and sudden cardiac death, which remains one of the most common causes of mortality among dialysis patients. Together, these observations highlight that nutritional status influences virtually every stage of cardiovascular disease, from endothelial injury and vascular calcification to myocardial remodeling and heart failure, making nutritional assessment and intervention an integral component of cardiovascular risk management in the dialysis population [8,9,43].

## 8. Mortality

Malnutrition is one of the strongest and most consistent predictors of mortality in patients receiving dialysis. While numerous nutritional assessment tools have demonstrated prognostic value, they capture different aspects of the complex syndrome of PEW [1,2,3,80]. Serum albumin remains the most extensively studied biomarker and is consistently associated with both all-cause and cardiovascular mortality. However, albumin should not be interpreted solely as a marker of nutritional status, as its concentration is also influenced by inflammation, fluid overload, hepatic synthesis, and acute illness. Consequently, contemporary guidelines recommend interpreting albumin within the broader clinical context rather than as an isolated nutritional indicator. Composite nutritional assessment tools, including the Geriatric Nutritional Risk Index, the Malnutrition–Inflammation Score (MIS, also known as the Kalantar Score), and the Subjective Global Assessment (SGA), provide superior prognostic information because they integrate anthropometric measurements, dietary intake, functional status, inflammatory burden, and clinical examination [1,2,3,4,5,80]. Similarly, the creatinine index, which serves as a surrogate marker of skeletal muscle mass and protein stores, has emerged as a robust predictor of long-term survival, with lower values consistently associated with increased mortality risk [4,31].

The relationship between PEW and mortality extends beyond statistical associations [79]. PEW affects virtually every physiological system required to survive acute and chronic illness. Loss of skeletal muscle impairs mobility and physical resilience, increasing the risk of falls, disability, respiratory failure, and prolonged recovery following illness or surgery. Simultaneously, nutritional deficiencies compromise innate and adaptive immunity, predisposing patients to severe infections, poor wound healing, and impaired recovery from hospitalization. Chronic inflammation, oxidative stress, endocrine disturbances, and micronutrient deficiencies further accelerate vascular injury, myocardial remodeling, and progressive cardiovascular disease. These interrelated mechanisms explain why malnourished patients experience substantially higher rates of infection-related, cardiovascular, and all-cause mortality [77,78,79].

Patients with PEW are less able to withstand intercurrent illness, hospitalization, or surgical procedures, often entering a self-perpetuating cycle in which inflammation, infection, hospitalization, and further nutritional decline reinforce one another. Frailty, sarcopenia, and immunosenescence frequently coexist with PEW, further reducing resilience and increasing vulnerability to adverse outcomes [81,82,83,84]. Thus, PEW should be viewed not simply as a nutritional disorder but as a systemic clinical syndrome that reflects profound biological dysregulation. Its association with mortality is therefore biologically plausible, multifactorial, and consistently observed across diverse dialysis populations, regardless of the nutritional assessment tool employed [4,5,6,78,79,80].

## 9. Physical Function, Cognitive Function, and Quality of Life

Patients receiving dialysis may experience progressive declines in physical function and muscle mass, particularly in the presence of PEW. Loss of skeletal muscle mass and strength may contribute to reduced exercise capacity, impaired mobility, and limitations in functional independence [84,85,86]. Muscle weakness is further exacerbated by chronic inflammation, metabolic acidosis, endocrine abnormalities, and physical inactivity, all of which contribute to declining functional status. As muscle strength deteriorates, patients become increasingly vulnerable to frailty, a multidimensional syndrome characterized by reduced physiological reserve and diminished resilience to stressors. Frailty and PEW frequently coexist and amplify one another, creating a vicious cycle of inactivity, muscle loss, and worsening nutritional status [85,86,87,88].

The clinical consequences of impaired physical function are substantial. Sarcopenia and frailty are strongly associated with slower gait speed, poor balance, and impaired performance on objective functional tests, translating into a higher risk of falls, fractures, disability, and loss of independence. These complications are particularly important in the dialysis population, where mineral and bone disorders, vitamin D deficiency, and reduced muscle mass collectively increase fracture risk and delay recovery following injury [85,86]. Functional decline also limits participation in rehabilitation programs and physical activity, further promoting muscle wasting and increasing dependence on caregivers. Consequently, preservation of nutritional status is increasingly recognized as a cornerstone of strategies aimed at maintaining mobility, preventing disability, and improving healthy aging among patients with ESKD [89,90].

Cognitive impairment is common among patients receiving dialysis and is multifactorial, with potential contributions from vascular disease, cerebral hypoperfusion, uremic factors, aging, comorbidities, and dialysis-related hemodynamic changes. The specific contribution of nutritional impairment remains incompletely understood. Chronic systemic inflammation -has been proposed as a potential contributor to neuroinflammation and endothelial dysfunction, whereas vascular disease and intradialytic hemodynamic instability may reduce cerebral perfusion, contributing to ischemic brain injury and progressive cognitive decline. Together, these factors likely accelerate deficits in attention, executive function, memory, and processing speed, all of which are highly prevalent in the dialysis population [91,92].

Quality of life is closely linked to nutritional status and represents one of the most meaningful patient-centered outcomes in dialysis care. Patients with PEW consistently report greater fatigue, lower physical functioning, reduced participation in daily activities, and higher rates of depressive symptoms compared with well-nourished individuals. Fatigue, one of the most common and debilitating symptoms experienced by dialysis patients, is driven by the combined effects of muscle wasting, inflammation, anemia, and reduced exercise tolerance. As nutritional status deteriorates, patients often become progressively less physically active, leading to further deconditioning, worsening sarcopenia, and social isolation. Frequent hospitalizations related to infections, cardiovascular events, or complications of frailty further disrupt daily life and contribute to declining health-related quality of life [93].

These relationships highlight that the consequences of malnutrition extend far beyond traditional biochemical or anthropometric measures. By influencing physical capacity, cognitive performance, emotional well-being, and the ability to live independently, PEW has a profound impact on patient-centered outcomes. Early identification of nutritional impairment and comprehensive multidisciplinary interventions are important components of supportive care. However, the extent to which nutritional interventions independently improve survival, functional outcomes, cognitive function, or quality of life requires further investigation.

## 10. Future Directions in the Understanding and Management of Malnutrition in Dialysis

Despite substantial advances in the characterization of PEW in dialysis patients, major gaps remain in early detection, mechanistic understanding, and individualized treatment. Emerging technologies and systems-level approaches are now reshaping the field, shifting it from largely observational and phenotype-based assessment toward precision-driven, data-integrated care. These developments hold promise for earlier identification of at-risk patients, improved risk stratification, and more targeted nutritional and metabolic interventions.

Artificial intelligence (AI) and machine learning are increasingly being applied to large clinical datasets in nephrology, enabling the integration of routinely collected variables, laboratory parameters, dialysis adequacy metrics, comorbidities, and outcomes to generate predictive models for malnutrition, hospitalization, and mortality. Explainable machine-learning models incorporating routine laboratory and clinical variables have recently demonstrated strong discriminative performance for identifying PEW risk in patients on maintenance hemodialysis [94,95]. AI-based approaches may potentially facilitate longitudinal risk assessment, but their clinical utility in nutritional evaluation among patients receiving maintenance dialysis remains to be established. This transition from point-in-time scoring systems (such as SGA or MIS) to dynamic predictive analytics could potentially support earlier identification of patients at risk, pending prospective validation and demonstration of clinical utility. Current evidence remains limited, and external validation and prospective studies are required before AI-based tools can be incorporated into routine clinical assessment.

Although predominantly investigational at present, omics technologies are providing new insights into the biological underpinnings of PEW. Metabolomics and proteomics allow comprehensive profiling of metabolic and protein signatures associated with inflammation, catabolism, and energy imbalance, potentially identifying novel biomarkers that outperform traditional nutritional indicators [96]. Similarly, microbiome research has highlighted the role of gut dysbiosis, endotoxemia, and microbial-derived metabolites in driving inflammation and metabolic disturbances in ESKD, and in perpetuating the immunosenescence that accompanies advanced kidney failure [97,98]. These approaches collectively may ultimately contribute to the development of more individualized approaches to nutritional assessment and intervention, in which dietary and pharmacologic interventions are tailored to an individual’s metabolic, inflammatory, and microbial profile rather than relying on generalized dietary recommendations.

Digital health tools, including wearable devices and remote monitoring systems, further expand the potential for real-time assessment of functional and nutritional status. These can provide continuous data on physical activity, sleep patterns, and physiological parameters such as heart rate variability, offering objective markers of frailty and functional decline [99,100]. These technologies may provide longitudinal information on physical activity and other functional parameters; However, their specific role in nutritional assessment and management in dialysis populations remains to be established.

Collectively, these innovations define a future in which the management of malnutrition in dialysis evolves from reactive treatment of established PEW to proactive, personalized prevention strategies. By integrating AI, omics-based biomarkers, microbiome profiling, dialysis data, and digital health technologies, nephrology is moving toward a precision nutrition paradigm aimed at improving survival, preserving function, and enhancing quality of life in this highly vulnerable population.

## 11. Conclusions

Malnutrition and PEW are common and clinically important complications of dialysis and are associated with immune dysfunction, infection, cardiovascular disease, frailty, impaired physical and cognitive function, reduced quality of life, and increased mortality. Although these associations are supported by a substantial body of observational evidence, the available data do not establish malnutrition as an independent causal driver of these outcomes, and residual confounding and reverse causation cannot be excluded. Malnutrition should therefore be considered a multifactorial marker and potential contributor to multisystem vulnerability in patients receiving dialysis. Comprehensive nutritional assessment may have an important role in risk stratification and clinical management; however, further prospective and interventional studies are needed to determine whether targeted strategies to prevent or treat malnutrition can modify these outcomes and improve patient-centered and clinical outcomes.

## Figures and Tables

**Figure 1:**
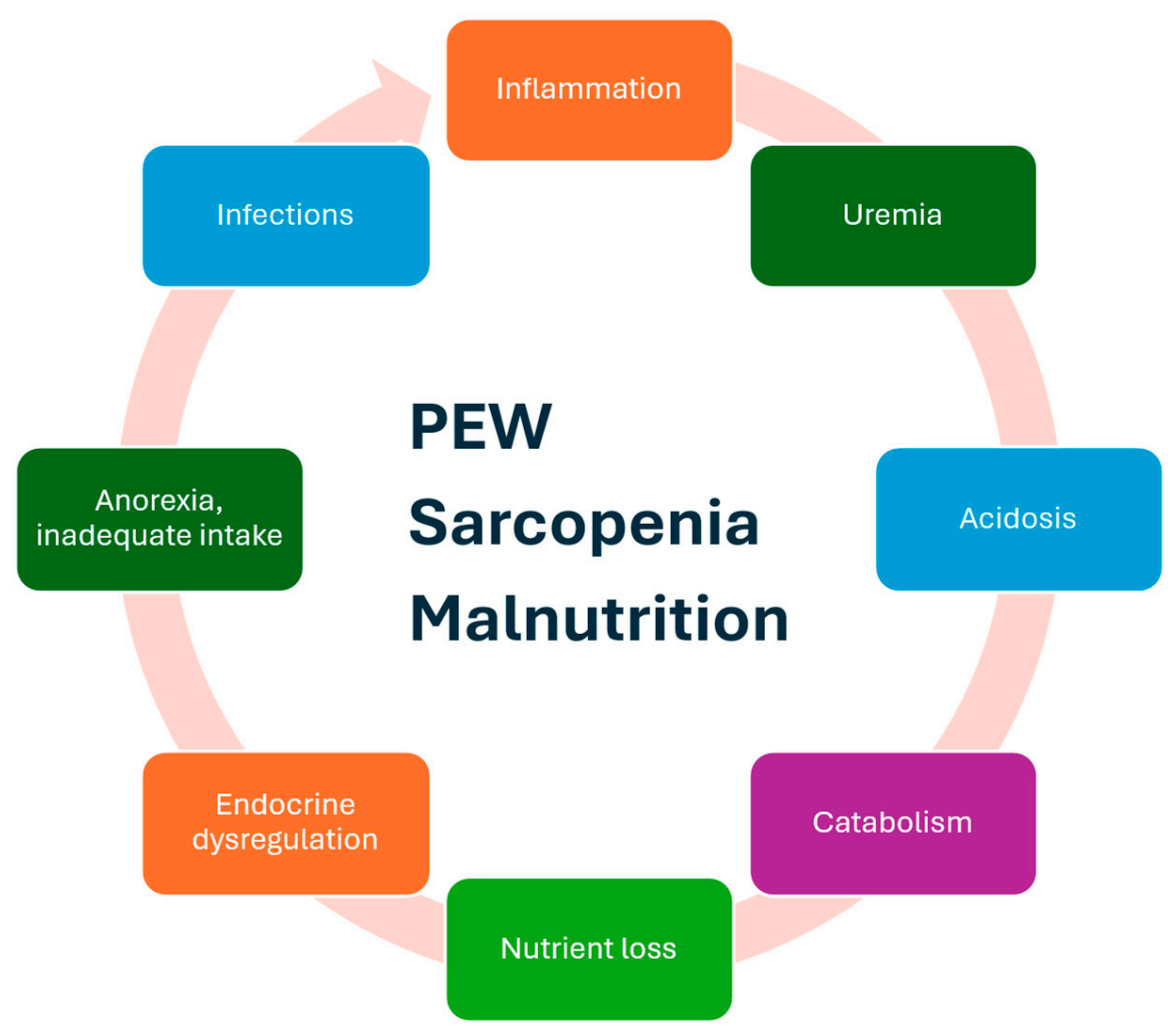
The multifactorial vicious cycle of malnutrition in dialysis patients. PEW, protein-energy wasting.

**Table 1:** Key characteristics and diagnostic frameworks of nutritional and functional syndromes in patients with kidney failure.

Condition	Primary Pathophysiology	Clinical Features
Malnutrition	Nutrient depletion resulting strictly from inadequate dietary protein/energy intake relative to demands	GLIM Criteria: (reduced food intake or assimilation,). Requires at least one phenotypic criterion (unintentional weight loss, low BMI, or reduced muscle mass) and one etiologic criterion (reduced dietary intake or malabsorption or disease burden/inflammation)
Protein-Energy Wasting (PEW)	CKD-specific loss of protein and energy stores; immunometabolic syndrome driven by uremic toxins, chronic systemic inflammation, hypercatabolism	ISRNM Consensus Framework: Requires satisfying criterion in at least 3 of the 4 categories: 1. Serum chemistry: serum albumin < 3.8 g/dL, serum prealbumin < 30 mg/dL, or serum cholesterol < 100 mg/dL. 2. Body mass: BMI < 23 kg/m^2^, unintentional weight loss of ≥5% over 3 months (or ≥10% over 6 months), or total body fat percentage < 10%. 3. Muscle mass: muscle wasting of ≥5% over 3 months (or ≥10% over 6 months), reduced mid-arm muscle circumference (>10% below the 50th percentile reference), reduced appendicular skeletal muscle index by DEXA, or low creatinine appearance. 4. Dietary intake: unintentional low dietary protein intake < 0.8 g/kg/day for at least 2 months in patients on dialysis (or <0.6 g/kg/day in earlier-stage CKD), or low dietary energy intake < 25 kcal/kg/day for at least 2 months.
Cachexia	Extreme, severe form of PEW driven by intense systemic inflammation, cytokine activation and uncompensated catabolism	Severe involuntary weight loss with profound skeletal muscle and fat wasting
Sarcopenia	Loss of muscle mass, quality, and physical strength	Low muscle quantity and quality
Frailty	Multisystem decline and reduced physiological reserve	Clinical Frailty Scale (CFS): judgment-based 9-point scale of fitness/frailty; slow walking speed, low handgrip strength, exhaustion, low physical activity, unintentional weight loss

## Data Availability

No new data were created or analyzed in this study. Data sharing is not applicable to this article.
